# Sludge disinfection using electrical thermal treatment: The role of ohmic heating

**DOI:** 10.1016/j.scitotenv.2017.09.175

**Published:** 2018-02-15

**Authors:** Ziqiang Yin, Michael Hoffmann, Sunny Jiang

**Affiliations:** a Department of Civil and Environmental Engineering, University of California, Irvine, Irvine 92617, CA, United States; b Division of Engineering and Applied Science, California Institution of Technology, Pasadena 91125, CA, United States

**Keywords:** AC, alternate current, DC, direct current, DI, deionized water, EEC, effective electrical conductivity, EMT, effective medium theory, EPA, environmental protection agency, LB, Luria-Bertani, ME, Maxwell–Eucken, MPN, most probable number, OH, ohmic heating, PBS, phosphate buffer saline, RMSD, root mean squared deviation, Sewage sludge, Energy efficiency, Pathogen inactivation, *E. coli*, Maxwell-Eucken model, Effective medium theory

## Abstract

Electrical heating has been proposed as a potential method for pathogen inactivation in human waste sludge, especially in decentralized wastewater treatment systems. In this study, we investigated the heat production and *E. coli* inactivation in wastewater sludge using electrical thermal treatment. Various concentrations of NaCl and NH_4_Cl were tested as electrolyte to enhance conductivity in sludge mixtures. At same voltage input (18 V), sludge treated with direct current (DC) exhibited slower ascent of temperature and lower energy efficiencies for heat production comparing to that using alternate current (AC). However, DC power showed better performance in *E. coli* inactivation due to electrochemical inactivation in addition to thermal inactivation. Greater than 6log_10_ removal of *E. coli* was demonstrated within 2 h using 0.15 M of NaCl as electrolyte by AC or DC power. The heat production in sludge was modeled using Maxwell–Eucken and effective medium theory based on the effective electrical conductivity in the two-phase (liquid and solid) sludge mixtures. The results showed that the water and heat loss is a critical consideration in modeling of sludge temperature using ohmic heating. The experimental data also suggested that the models are less applicable to DC power because the electrochemical reactions triggered by DC reduce the concentration of NH_4_^+^ and other ions that serve as electrolyte. The results of this study contribute to the development of engineering strategies for human waste sludge management.

## Highlights

• The relationship of ohmic heating and sludge condition was investigated.
• Solid fraction and conductivity are two critical factors for heat production.
• Heat production in sludge was successfully predicted using theoretical models.
• AC power is more effective to heat sludge than DC power.
• Ohmic heating is energy efficient for decentralized sludge disinfection.

## Graphical abstract

**Figure:**
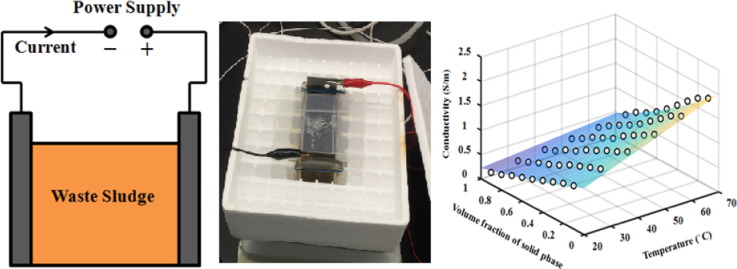

## 1 Introduction

Worldwide, 2.7 billion people rely on onsite sanitation. Yet, there is still no adequate management system in place to deal with the resulting fecal sludge. The Reinvent the Toilet Challenge initiated by Bill and Melinda Gates Foundation in 2011 has effectively promoted the creation of a toilet that removes germs from human waste; operates without connections to water, sewer or electrical lines; and promotes sustainable and financial profitable sanitation services. The solar toilet developed by California Institute of Technology emerged as a promising technology that has demonstrated efficiency and reliability in both lab-scale and field-scale operation to removal human pathogens from wastewater (Huang et al., 2016, CalTech News, n.d). The technology relies on electrochemical oxidation of organics and pathogens in the outflow of waste settling tank to generate treated water for reuse in toilet flushing. However, the current technology is not suitable for waste sludge treatment in the storage tank due to the high concentration of organics and associated energy demand for their removal.

Direct disposal of waste sludge, even after partial digestion, contaminates water and soil with high loads of human pathogens (Sidhu and Toze, 2009, Guzman et al., 2007). Diarrheal diseases and soil-transmitted helminthes infections are common occurrence among children in developing countries and are significant burdens to quality and expectancy of life. On the other hand, the nutrients and biomass contained in waste sludge could potentially be useful for enhancing agriculture production (Lu et al., 2012, Mateo-Sagasta et al., 2015). Sludge application to land as soil amendments or fertilizers is commonly practiced in many countries (Lowman et al., 2013, Yang et al., 2015, Pritchard et al., 2010, Lyberatos et al., 2011), yet often without the proper consideration of human health risk from exposure to pathogens.

In the developed country such as United States, pathogen inactivation is mandated prior to sludge disposal or land application (U.S. Environmental Protection Agency, 2003). A number of treatment methods have been authorized by the US EPA for sludge management, including composting, lime stabilization, aerobic digestion and anaerobic digestion. Aerobic digestion and anaerobic digestion are perhaps the most common methods for on-site treatment systems, but disinfection of pathogens is very slow in such processes. For example, it may take 60 days to reduce bacterial and viral pathogens at room temperature, while parasites such as helminthes ova may still be active after treatment (U.S. Environmental Protection Agency, 2003). Disinfection methods such as chlorination, radiation or ozonation that are commonly applied to wastewater effluent are not suitable for sludge treatment. Development of effective technology for onsite sludge disinfection is therefore necessary and urgent.

Ohmic heating (OH), an electrical thermal treatment, may have the potential to serve as the technology for onsite sludge disinfection. OH is produced by electric current passing through materials. It has a long and successful history of application in the food industry for food sterilization (Chen and Mujumdar, 2002, Tuan et al., 2012, Zhan et al., 2015, Daneshmand et al., 2012) and is more recently proposed for disinfection of sewage sludge (Takhtehfouladi et al., 2013, Takhtehfouladi et al., 2015). Comparing to traditional external heating processes, OH can rapidly and uniformly increase the temperature of target material without the need of heat transfer between the solid-liquid interface because heat is produced within the material (De Alwis and Fryer, 1992, Knirsch et al., 2010, Sastry and Barach, 2000, Sakr and Liu, 2014). Temperature is considered as a key measure in pathogen inactivation during OH processes, and it is positively related to the effective electrical conductivity (EEC) of the target specimen (Sastry and Palaniappan, 1992, Palaniappan and Sastry, 1991).

Determination of EEC in a multi-phase mixture (i.e. chicken soup or waste sludge) is perhaps the most important but challenging step in understanding the electrical treatment efficiency. Comparison of several previously published models for EEC (Mahmoud et al., 2011, Han and Choi, 1998) indicates that Maxwell–Eucken (ME) and effective medium theory (EMT) model are the best accepted models for predicting EEC of two-phase food mixture during OH treatment (Perez and Calvelo, 1984, Zhu et al., 2010). The ME model considers the mixture as a heterogeneous medium that contains one or more dispersed phases embedded in a continuous phase (Pietrak and Wisniewski, 2015, Wang et al., 2008). It usually includes two forms: 1) liquid phase serves as the continuous phase in ME-1 model; and 2) solid phase serves as the continuous phase in ME-2 model. By contrast, the EMT model is used to estimate the EEC when there is no obvious dispersed phase or continuous phase. It is a generalized model with the assumption that all phases in a heterogeneous medium are randomly distributed and mutually dispersed (Wang et al., 2006, Wang et al., 2008). Neither model has been applied to electrical heating in sludge treatment to predict thermal conductivity and heat production.

In the past, applications of electric field in sludge treatment mainly focused on sludge dewatering (Glendinning et al., 2009, Mahmoud et al., 2010, Tuan et al., 2012, Citeau et al., 2012). By applying direct current (DC) through sludge, water is separated from sludge particles by electro-osmotic force with relatively low energy consumption (Zhan et al., 2015, Esmaeily et al., 2006, Habel, 2010, Huang et al., 2008, Navab-Daneshmand et al., 2012). OH can enhance the electro-dewatering processes by increasing the sludge temperature, evaporation and electro-osmotic flow (Mahmoud et al., 2011, Mahmoud et al., 2016, Navab-Daneshmand et al., 2015). Recently, a Bioelectro technology was proposed for sludge/biosolid disinfection, in which OH was implied in the heat production (Takhtehfouladi et al., 2013, Takhtehfouladi et al., 2015). Sludge temperature reached 95 °C within a few hours at an applied voltage gradient of < 5 V/cm (Takhtehfouladi et al., 2013). Electric conditioners, such as ammonia salts were added to increase conductivity of sludge mixture and promote heat production (Esmaeily et al., 2006, Safaei et al., 2009). The reliable performance in inactivating a wide range of microorganisms in sludge through electrical heating process was demonstrated and was proposed as sustainable approach for waste sludge management (Esmaeily et al., 2006, Habel, 2010, Huang et al., 2008, Takhtehfouladi, 2007, Takhtehfouladi et al., 2013).

Navab-Daneshmand et al. (2012) suggested that OH was the primary mechanism for microbial inactivation in biosolids during electro-dewatering treatment. Other studies demonstrated that the formation of chemical byproducts, such as chlorine or H_2_O_2_ during electric thermal treatment facilitated pathogen removal (Huang et al., 2016, Habel, 2010, Li et al., 2004). The contribution of these inactivation mechanisms at different operational conditions needs to be further investigated and clarified. Furthermore, the heat production and energy consumption vary with volume fraction of solid sludge, the concentration of inorganic and organic salts in the sludge mixture and type of current (AC vs. DC) applied (Zhang et al., 2017, Habel, 2010, Esmaeily et al., 2006, Takhtehfouladi, 2012). The operational parameters of the electrical thermal treatment should be carefully designed, controlled and optimized in order to reach energy efficiency and complete inactivation of pathogens. The objectives of this study are to: 1) compare and optimize the operational parameters, including different power supplies and salt concentrations, to achieve energy efficiency and pathogen removal in sludge treatment; 2) validate the ME and MET model in predicting the effective electrical conductivity of sludge; 3) develop a mathematical model to predict the heat production and temperature increase during the OH treatment. The study presented here offers mechanism understanding of heat production during OH and presents strategies for design optimization for enhancing the sludge treatment efficiency.

## 2 Material and methods

### 2.1 Sludge sampling and preparation

Secondary (returned) activated sludge was collected from Orange County Sanitation District (Fountain Valley, CA). The plant represents a typical large municipal wastewater treatment plant using activated sludge process as the secondary treatment. The typical mixed liquor suspended solid (MLSS) of the sludge ranges between 5000 and 10,000 mg/L. To investigate the influence of sludge condition such as salt concentration and solid fraction on the efficiency of sludge heat treatment, sludge samples were settled for 3 h and the supernatant was discarded. The settled sludge was washed with DI water and pelleted by centrifugation (Eppendorf Centrifuge 5810R) at 3500 rpm (2465 g) for 15 min. Sludge pellet was collected after repeating the washing procedure 3 times and used as the solid phase of sludge to investigate the relationship between applied electrical power and EEC (and associated sludge temperature) at different salt concentrations. By controlling the sludge solid fraction using general sludge property with added salts at different concentration, we could validate the applicability of existing models for heat production in sludge. Cultured *E. coli* was seeded in the sludge mixture in the disinfection experiments. All samples were kept at 4 °C until processing.

### 2.2 Experimental set-up

The reactor for sludge treatment used in this study consists of acrylic rectangular cell with interior dimension 9 cm × 4 cm × 5 cm, and two plate electrodes made of stainless steel. The reactor was placed in a plastic foam container to reduce heat loss, and the container was placed on a digital balance to record the change of weight as water evaporating during the experiments. DC power was provided by a Tekpower TP3005T power supply with maximum voltage output of 30 V, while AC power was generated by an Enercell, 18/24 V switchable AC power adapter. Electric current was measured by a digital multimeter (Sinometer® MAS-345). For all experiments, 18 V of either AC or DC power was applied.

### 2.3 EEC in sludge mixtures

NaCl and NH_4_Cl were used as electrolytes to model EEC in sludge. Sludge mixtures with solid volume fractions ranging from 0, 0.1, 0.2, 0.3, 0.4, 0.5, 0.6, 0.7, 0.8, 0.9 to 1.0 were prepared by mixing the sludge pellet and 0.1 M of NaCl or NH_4_Cl solution using formula (1):

(1) $$x_{S}=\frac{V_{S}}{V_{S}+V_{L}}$$

where, *x*_*S*_ is the solid volume fraction, *V*_*S*_ is the volume of centrifuged sludge pellet; *V*_*L*_ is the volume of salt solution. The use of solid volume fraction to indicate the thickness of the sludge is to be consistent with the model requirement for computing EEC in mixture.

Freshly prepared sludge mixture (100 mL) was mixed vigorously in beakers by vortexing at top speed for 15 s and heated in water bath at ~ 80 °C for 30 min before transferred into the reactor. In the reactor, the sludge was stirred periodically to mix and the temperature was monitored continuously. DI water was added according to the reading of balance to offset the water loss due to evaporation. When the temperature of sludge mixture dropped to 70 °C, 60 °C, 50 °C, 40 °C, 30 °C, and 20 °C, 18 V of AC power was applied on the reactor for a few seconds until the current reading on the multimeter was stable before collecting the current readings at each corresponding temperature. The corresponding EEC (*σ*) was calculated by

(2) $$\sigma =\frac{I}{U}∙\frac{l}{A}$$

where, I is the electric current, U is the electric voltage (18 V), *l* is the length of the reactor, A is the cross-section area of the reactor. Each test for a specific electrolyte was conducted in triplicate. DC power was not used to measure electric current in the sludge due to its potential to cause electrochemical reactions that could interfere with EEC.

To validate that EEC in sludge mixture can be estimated using theoretical ME or EMT model, experimental data collected from EEC experiments were compared with the predication from the models. The size/shape factor (d_i_) is an important parameter related to particle size and sphericity in the two-phase structural models. d_i_ = 3 was selected as in the previous studies (Zhu et al., 2010, Wang et al., 2006). Both ME-1 (Eq. (3)) and ME-2 (Eq. (4)) models were applied to the sludge mixture as by Zhu et al. (2010) as:

(3) $$\sigma =\frac{x_{L}∙\sigma_{L}+x_{S}∙\sigma_{S}∙\frac{3\sigma_{L}}{2\sigma_{L}+\sigma_{S}}}{x_{L}+x_{S}∙\frac{3\sigma_{L}}{2\sigma_{L}+\sigma_{S}}}$$

(4) $$\sigma =\frac{x_{L}∙\sigma_{L}∙\frac{3\sigma_{S}}{\sigma_{L}+2\sigma_{S}}+x_{S}∙\sigma_{S}}{x_{L}∙\frac{3\sigma_{S}}{\sigma_{L}+2\sigma_{S}}+x_{S}}$$

where the EEC (*σ*) of the mixture is a function of the volume fraction of liquid (*x*_*L*_) and solid (*x*_*S*_) phase, the EEC of pure liquid (*σ*_*L*_) and solid (*σ*_*S*_) phase. ME-1 and ME-2 model differ in the consideration of continuous phase in the two-phase mixture.

The EMT model assumes that in an electric field, the sum of deviated polarization of spherical inclusions in a homogeneous medium is zero (Choy, 2015), which can be expressed in Eq. (5) for a two-phase sludge mixture,

(5) $$x_{L}∙\frac{\sigma_{L}-\sigma }{\sigma_{L}+2\sigma }+x_{S}∙\frac{\sigma_{S}-\sigma }{\sigma_{S}+2\sigma }=0$$

The theoretical EEC curves at different solid volume fraction were computed by applying the measured conductivity at boundary conditions (*σ*_*L*_ at *x*_*S*_ = 0 and *σ*_*S*_ at *x*_*S*_ = 1) to each of the models above (Eqs. (3), (4), (5)). The EEC is converted to corresponding sludge temperature using Eq. (6) (Goullieux and Pain, 2005).

(6) $$\sigma_{T}=\sigma_{0}∙\left[1+a∙\left(T-T_{0}\right)\right]$$

where, *σ*_0_ and *σ*_*T*_ is the conductivity at the beginning *T*_0_ and temperature *T*, respectively; *a* is the temperature coefficient of variation, which were determined experimentally (Barron and Ashton, 2005). The predicated EEC was compared statistically with EEC measurements at various solid fractions of sludge mixtures for each temperature. The statistical analysis for model fitting was carried out using Matlab® (MathWorks Inc.).

### 2.4 Modeling energy usage and heat production

In order to understand the energy requirement for effective heat inactivation of pathogen, the electrical energy needed for heat production should be determined. According to the energy conservation equation (Eq. (7)), the electrical energy input (*Q*_*e*_) can be converted to heat in the substrate (*Q*_*heat*_), electrochemical reactions (*Q*_*chem*_), and be dissipated as heat loss to the environment (*Q*_*loss*_).

(7) $$Q_{e}=Q_{\mathrm{heat}}+Q_{\mathrm{chem}}+Q_{\mathrm{loss}}$$

DC electrical treatment is well known to generate electrochemical reactions in sludge (Chen et al., 2007, Li and Liu, 2009), while in comparison, electrochemical reactions using AC power are much reduced (Kamaraj et al., 2013, Fu and Cheng, 2010). To simplify the energy calculation, we assumed the net energy associated with electrochemical reactions (*Q*_*chem*_) was negligible when AC was applied.

Using applied voltage (*U*), the *Q*_*e*_ can be calculated by integration of substrate EEC (*σ*) over time and the volume in reactor (*V*) using Eq. (8), where *E* is the strength of local electric field.

(8) $$Q_{e}\left(t\right)=U∙I\left(t\right)∙t=E^{2}∙V∙\int_{0}^{t}\sigma \left(t\right)\mathrm{dt}$$

The amount of energy used for heat production (*Q*_*heat*_) to reach certain desired temperature (*T*) is given by Eq. (9).

(9) $$Q_{\mathrm{heat}}=m∙C_{p}∙\left(T-T_{0}\right)$$

where m is the mass of the sludge (in gram), *C*_*p*_ is the specific heat capacity of sludge mixture, *T*_0_ and *T* is the initial temperature and temperature at time t, respectively. The specific heat capacity (*C*_*p*_) of sludge mixture was experimentally determined using sludge pellet mixed with 0.05 M, 0.1 M or 0.15 M NaCl or NH_4_Cl solutions respectively at solid volume fraction (*x*_*S*_) of 0.2. Sludge mixture in triplicate tubes was heated in a 50 °C water bath for 3 min, and the temperature over time in each tube was recorded. The heat capacity of sludge mixtures (*C*_*p*_) was calculated using DI water as reference with the standard specific heat capacity of 4.18 J/g °C (USGS). Heat capacity for other solid volume fractions can be determined using the same approach.

Evaporation (*Q*_*ev*_) and heat dissipation (*Q*_*dis*_) are considered as the main cause of heat loss as shown in Eq. (10).

(10) $$Q_{\mathrm{loss}}=Q_{\mathrm{ev}}+Q_{\mathrm{dis}}$$

The heat loss due to water evaporation (*Q*_*ev*_) was calculated by Eq. (11).

(11) $$Q_{\mathrm{ev}}=m_{\mathrm{ev}}\left(t\right)∙L\;$$

where, *m*_*ev*_ is the mass of water evaporated, *L* is the specific latent heat of water evaporation 2264.76 kJ/kg (Lu et al., 2017). Heat dissipation (*Q*_*dis*_) is the amount of heat transferred from the sludge mixture to the ambient environment through thermal conduction. It can be computed using Eq. (12).

(12) $$Q_{\mathrm{dis}}=\left(1-\mathrm{InF}\right)∙\mu ∙A_{s}∙\left(T_{s}-T_{a}\right)∙t$$

where, *InF* is the insulation factor; *A*_*s*_ is the surface area for heat dissipation, and it was calculated according to the dimensions of the reactor; *T*_*s*_ and *T*_*a*_ are the sludge and ambient temperature, respectively. *μ* is the heat transfer coefficient, which can be determined by Eq. (13).

(13) $$\mu =\frac{1}{\frac{1}{\mu_{\mathrm{in}}}+\sum_{1}^{n}\frac{\delta_{i}}{k_{i}}+\frac{1}{\mu_{\mathrm{out}}}}$$

where, *μ*_*in*_ and *μ*_*out*_ are the heat transfer coefficients in and outside of the reactor, respectively; *δ*_*i*_ is the thickness of the reactor wall; *k*_*i*_ is the thermal conductivity of the wall. The heat transfer coefficient and thermal conductivity are related to the material property. The typical values of air heat transfer coefficient ranges from 10 to 100 W/m^2^ K (Engineering Tool Box 1). Water, on the other hand, possesses the typical heat transfer coefficient from 100 to 1200 W/m^2^ K in free convection (Engineers Edge). Generally, the heat transfer coefficient is positively correlated to the velocity of air or water flow over a surface (Engineering Tool Box, n.d, GE Power and Water, n.d). Since no specific mechanism to promote air or water flow was used in the experiments, lower end of heat transfer coefficients (10 W/m^2^ K for air and 100 W/m^2^ K for water) were selected from the respective range. The reactor used in this study was made of acrylic, for which the thermal conductivity is 0.2 W/m K (Engineering Tool Box 3). The parameters and constants used in the modeling are listed in Table S1.

### 2.5 Sludge treatment by OH for *E. coli* disinfection

The solid volume fraction (*x*_*S*_) of 0.2 was used in the OH sludge treatment experiments. Three concentrations of NaCl or NH_4_Cl (0.05 M, 0.1 M and 0.15 M) were used to determine the relationship between electrolyte concentration and heat production. For each experiment, 100 mL of sludge mixtures were transferred into the reactor, and 18 V of AC or DC power was applied. The experiments lasted for 4 h with 0.05 M and 0.1 M of salts, and the electric current and temperature data were recorded every 30 min during each experiment to determine the efficiency of OH in sludge. When 0.15 M of salts was used, each experiment was conducted for 2 h, and the data were collected every 15 min. A preliminary experiment with DI water only showed no significant heat production in the absence of salts.

*E. coli* inactivation tests were conducted using sludge mixture containing 0.15 M NaCl or 0.15 M NH_4_Cl and seeded *E. coli K-12* (ATCC 10798). The single colony of *E. coli* was incubated overnight in Luria-Bertani (LB) broth at 38 °C. *E. coli* cells were pelleted by centrifuge at 3500 rpm (2465*g*) for 10 min, and re-suspended with phosphate buffer saline (PBS) solution. The fresh cell suspension was seeded in the sludge reactor for quantification of *E. coli* inactivation by subsampling treated sludge over time.

EPA method 1680 (U.S. Environmental Protection Agency, 2010) was employed for quantification of *E. coli*. Briefly, at least 4 serial dilutions were made for each *E. coli* sample collected from sludge reactor using PBS, and 5 replicates were made for each dilution. Diluted *E. coli* samples were aliquot into test tubes containing sterilized lauryl tryptose broth (LTB, Remel Microbiology) with an inverted Durham tube (VWR). After incubating at 35 °C for 24 ± 2 h, tubes with both turbidity and gas production were picked as presumptive positive tubes. Other tubes were incubated at 35 °C for an additional 24 ± 2 h, and considered as negative tubes if no turbidity and gas production was observed afterward. The growth from each presumptive positive tube was spiked into fresh tubes with sterilized EC medium (Remel Microbiology), and incubated in a 44.5 °C water bath for 24 ± 2 h. Tubes with both turbidity and gas production were recorded as positive reactions. Three significant dilutions with positive reactions were selected, and the concentration (in Most Probable Number, MPN) of *E. coli* was calculated accordingly.

Disinfection experiment were carried out using 18 V of AC or DC power for 2 h. Sampling for *E. coli* quantification were conducted every 15 min. At each sampling point, sludge was mixed, and 0.2 mL of sludge sample was collected from the reactor then diluted with 1.8 mL PBS solution. All samples were processed for *E. coli* test within 24 h after sampling.

## 3 Results

### 3.1 Prediction of EEC using ME model and EMT model

**Fig. 1:**
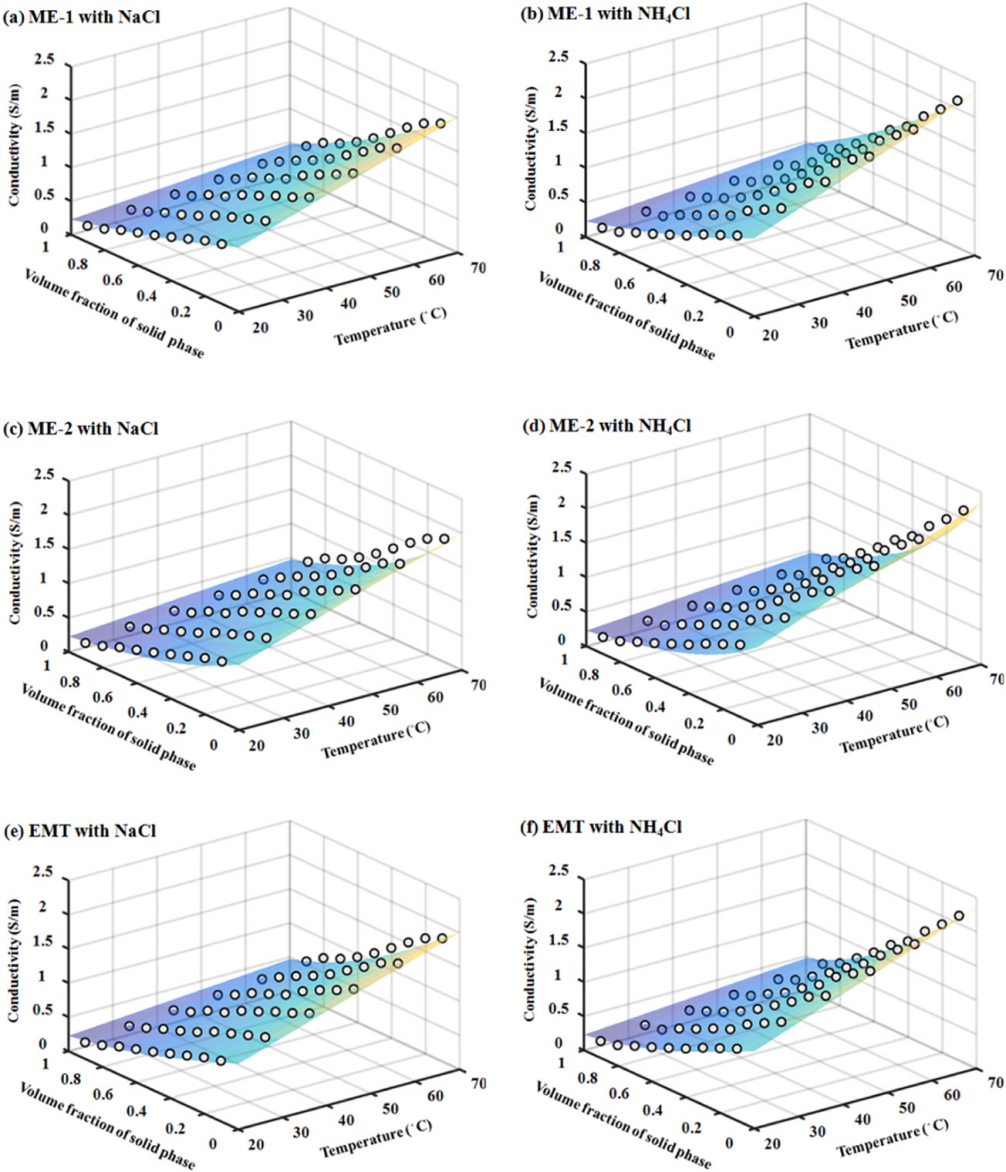
Plots of EEC values predicted by different models (surface) against measured average data (dots) at different temperature and sludge volume fractions using 0.1 M NaCl and 0.1 M NH_4_Cl as electrolyte, respectively.

The predicted EEC by ME-1, ME-2, and EMT models are plotted together with the measured data in Fig. 1. It can be seen that the EEC of sludge mixture with NaCl or NH_4_Cl as electrolyte showed near linear decline with the increase of solid fraction in the sludge. At the same temperature and volume fraction, sludge mixtures with 0.1 M NH_4_Cl had higher EEC comparing to those with 0.1 M NaCl. Linear and positive correlations (r^2^ > 0.99) between temperature and EEC were observed regardless of volume fractions and types of salt for all experimental data, agreeing with previous reports in various types of two-phase mixtures, including sludge mixtures (Icier and Ilicali, 2005, Murphy et al., 1991, Zhu et al., 2010, Sarang et al., 2008, Palaniappan and Sastry, 1991).

The model predicted EEC also agrees reasonable well with experimental measured EEC in all settings. The statistical analysis of model and experimental data indicated that the coefficients of determination (r^2^) of all models were > 0.9, suggesting strong fits between the predicted and measured data (Table 1). Comparing the three models applied, ME-2 was relatively weaker than the other two models as indicated by the lower r^2^ and greater root mean squared deviation (RMSD). Model fitting for data collected only from high solid fraction experiments (0.5 ≤ *x*_*S*_ ≤ 0.9) also carried out to determine if ME-2 model was better suited for high solid fraction mixture since it considers solid as the continuous phase. However, the outputs again indicated that ME-2 was weaker comparing to ME-1 and EMT (data not shown). Since the ME model and EMT model were nonlinear models, the coefficients of determination (r^2^) may not be a reliable indicator for model fit. Additional model validation using scatter plot of observed data against predicted values showed that the correlation coefficients (R^2^) were > 0.95 (Table 1). When the intercepts were deliberately set to zero, the slope of predicated vs. measured data were compared. The outcome again confirmed that the performance of ME-1 and EMT model were better than ME-2, in which the slopes were closer to 1 comparing to the ones produced by ME-2.

**Table 1:** Summary of key parameters in statistic analysis.

Model	Salt	Regression equation of scatter plot	Correlation coefficient (R^2^)	RMSD	Coefficient of determination (r^2^)	*p*-Value (residual analysis)
ME-1	NaCl	y = 1.006x	0.9823	0.051	0.982	< 0.05
	NH_4_Cl	y = 0.9677x	0.9752	0.081	0.970	0.81
ME-2	NaCl	y = 1.115x	0.9711	0.12	0.907	< 0.05
	NH_4_Cl	y = 1.1125x	0.9748	0.128	0.924	< 0.05
EMT	NaCl	y = 1.0556x	0.9655	0.057	0.949	< 0.05
	NH_4_Cl	y = 1.0062x	0.9868	0.054	0.987	0.67

R^2^ is the correlation coefficient of the scatter plot (*p* < 0.05); r^2^ is the coefficient of determination between the model and experimental data.

It should be pointed out that the residuals calculated based on experimental data and the predicted values from ME and EMT models failed to pass the normality test (*p* < 0.05) in two settings (Table 1), indicating these residuals were not randomly distributed. The non-randomly distributed residuals generally would not become a fatal issue, but it attenuated the consistency of the model fitting. Generally, the results of statistic analysis suggested that the ME and EMT models can be used to predict the EEC in sludge mixture, and the performance of ME-1 and EMT are superior to ME-2. Therefore, the ME and EMT should be used for estimation of heat production for OH treatment of sludge.

### 3.2 Effect of electrolyte and power on sludge temperature

**Fig. 2:**
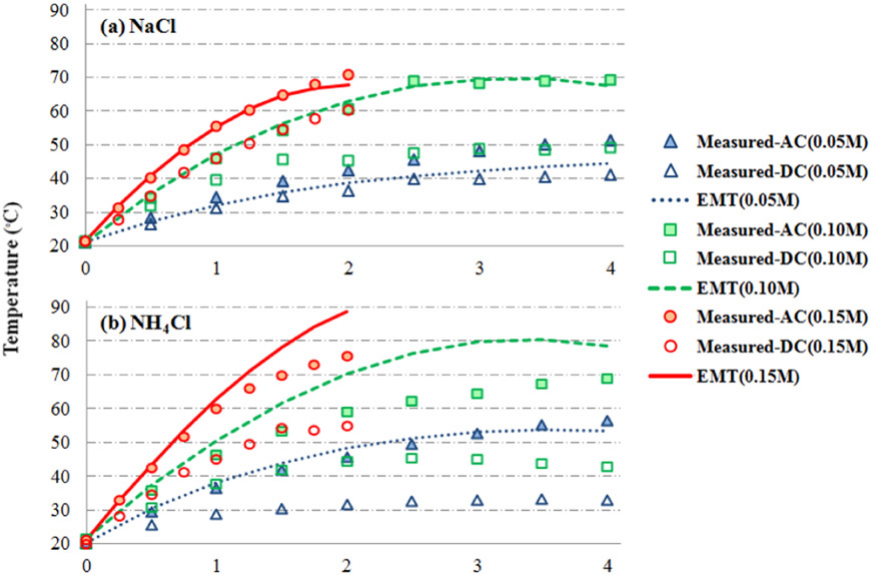
Measured and predicted temperature during ohmic heating treatment (InF = 0.65).

As shown in Fig. 2, the rise of temperature was faster in sludge mixtures with higher salt concentration. In sludge mixtures with 0.15 M NaCl or NH_4_Cl, the temperature exceeded 70 °C within 2-hour operation with AC power at 18 V. In comparison, if the sludge contained 0.05 M of salt, the temperature only reached 50 °C to 55 °C after 4 h. NH_4_Cl was more effective in enhancing heat production since it has higher EEC in nature comparing to NaCl at same concentration.

When the power supply switched to DC at 18 V, the rate of temperature increases in sludge mixtures was slower in general comparing to AC. For example, the temperature of sludge mixture with 0.15 M NaCl and NH_4_Cl went up to 60.5 °C and 55.0 °C respectively after 2-hour heating (Fig. 2). If the salt concentrations were lowered to 0.05 M, the temperature of sludge only elevated to 41.3 °C and 33.3 °C at the end of 4-hour experiments. The temperature of sludge with NaCl increased faster than that with the same concentration of NH_4_Cl, which was opposite to the phenomenon observed with AC power (Fig. 2). The EEC predicted by the ME-1 model and the EMT model, as well as the calculated temperature, was very similar to each other, and only the temperature using predicated EEC from the EMT model are shown in Fig. 2 for comparison (*Q*_*chem*_ was assumed to be zero). The predicted values were best matched with the measured temperature using AC power. The measured sludge temperatures were lower than the model predictions at higher temperatures due to heat lost from the sludge during treatment process (Fig. 2).

### 3.3 Energy usage for heat production

**Fig. 3:**
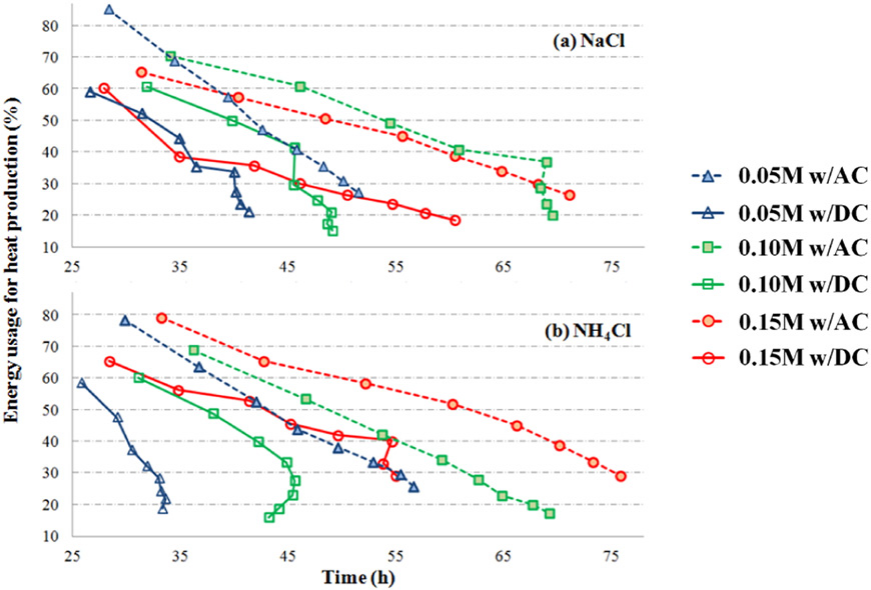
Percentage of energy usage for heat production at different experimental conditions.

The specific heat capacity (*C*_*p*_) of sludge mixture at *x*_*s*_ = 0.2 with NaCl and NH_4_Cl was determined as 3.49 J/g °C and 3.55 J/g °C, respectively. Accordingly, approximate 17.5 KJ of energy is needed to heat 100 mL of such sludge to 70 °C based on Eq. (9). During the electrical treatment of sludge, the electricity energy may also be consumed by the electrochemical reactions in sludge or on electrodes, or dissipated to the environment. Fig. 3 showed the plots of energy usage for heat production vs. sludge temperature during experiments with different power sources and electrolytes. The energy efficiency decreased with increase of sludge temperature. Energy efficiency for heat production in sludge with AC power was obviously higher than that with DC power, regardless of type of electrolyte and concentration. Higher concentration of salt generally led to greater energy efficiency of heat production.

### 3.4 Inactivation of *E. coli*

**Fig. 4:**
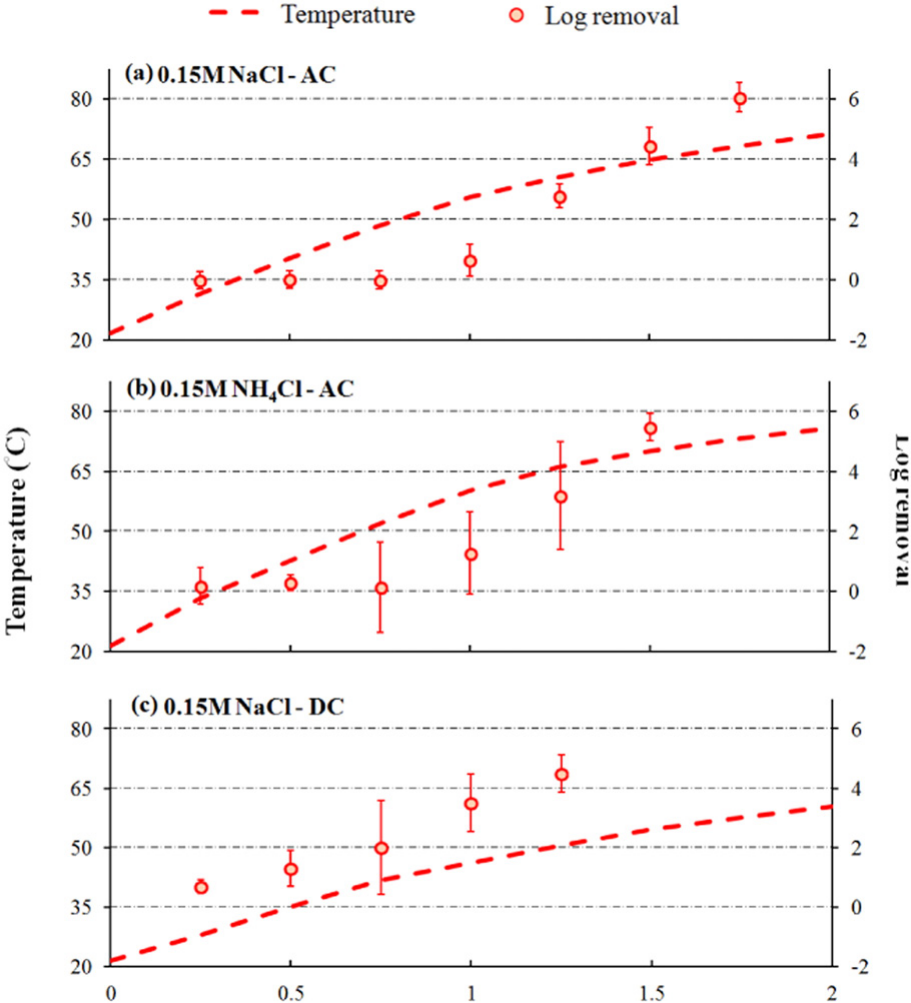
Temperature and removal of *E. coli* over treatment time.

Results of *E. coli* inactivation test are summarized in Fig. 4. Using AC power, *E. coli* concentration remained almost unchanged during the first 1 h in sludge mixtures with 0.15 M NH_4_Cl or 0.15 M NaCl. In sludge mixture with NH_4_Cl, the temperature increased by 10 °C from 1 h to 1.5 h, while the average removal of *E. coli* increased from 1.3 to 5.5 log_10_. The *E. coli* concentration declined to below the detection limit (0.3 log_10_) after 1.75 h, as the sludge temperature reached 70 °C. In contrast, *E. coli* inactivation was roughly half log_10_ lower in sludge mixture with NaCl for the similar time period. The removal was 2.7, 4.4 and 6.0 log_10_ at 1.25 h, 1.5 h and 1.75 h, respectively, and no *E. coli* was detected after 2 h. When it came to DC power, the concentration of *E. coli* decreased steadily throughout the experiment. The removal was 0.68, 2.0, and 4.5 log_10_ at 0.25 h, 0.75 h and 1.25 h, as the temperature rose to 28 °C, 42 °C, and 50 °C, respectively. The *E. coli* concentration was below detection limit in the rest of the experiment.

## 4 Discussion

### 4.1 Model implications

The data from our experiments suggest that the two existing models, ME-1 and EMT, are able to accurately predict the EEC in sludge mixtures, and the predictions from these two models are similar to each other. Determination of the solid volume fraction and the conductivity of the liquid and solid phase in sludge mixture are the key steps to apply the models. The conductivity of the liquid phase relies on the ionic strength, which could vary with the wastewater quality. Sludge conductivity and temperature are two interdependent parameters in OH treatment. Higher temperature generally leads to greater conductivity. While with constant voltage input, higher conductivity will further enhance the heat production.

A “porous-plug” model, initiated by Wyllie and Southwick (1954), has been successfully used to estimate the EEC in wastewater sludge and other two-phase mixtures (Mahmoud et al., 2006, Mahmoud et al., 2010, Zhang et al., 2017). It assumes three pathways when the electrical current passes the mixture, including the alternating layers of particles and interstitial liquid, contiguous particles and continuous liquid phase. A total of five parameters need to be determined experimentally in order to apply the “porous-plug” model, and they are affected by the properties of the mixture. Comparatively, the ME and EMT models are simpler, and the model parameters (e.g. volume fraction, conductivity of single phase) are easy to determine as we have described in this study. However, the two models may lose accuracy if the distribution of sludge particles (solid phase) is polarized due to electro-osmosis or gravitational settlement.

The energy model developed in this study is based on the energy conservation theory, and thus estimation of heat loss is critical to the model accuracy. Evaporation is one of the major pathways of heat loss, and the simultaneous loss of water may also affect the volume fraction and the conductivity in each phase of the sludge. In our study, the reactor was placed on a balance so the mass of sludge was recorded, and the water loss could be calculated. However, the measurement of sludge weight may not be applicable in practice. In this case, the evaporation rate from water surfaces may be used. Evaporation rate has been extensively studied with a number of established models (Monteith, 1981, Zotz and Thomas, 1999, Talati, 1988). If DC is selected as power source, additional water loss due to water electrolysis may need to be considered (Mahmoud et al., 2010, Mahmoud et al., 2016). Heat dissipation through thermal conduction is another major cause of heat loss. Our experimental results indicated that approximate 70%–80% of energy was lost as the temperature of sludge reached 70 °C (Fig. 3), and the model suggests that heat preservation is the key to minimize energy consumption in OH treatment. For example, by increasing the insulation factor from 0.35 to 0.5, the time and electrical energy predicted to heat the sludge with 0.1 M NaCl to 70 °C will be reduced by around 40%.

### 4.2 Impact of salt concentration

Salt concentration is a key parameter controlling electrical conductivity and heat production in sludge during OH treatment. As shown in Fig. 2, it is clear that higher levels of salts resulted in faster rise of temperature. The growth of conductivity and temperature was much slower when the treatment was driven by DC power, especially in the sludge with addition of NH_4_Cl (Fig. 2 and Fig. S1). Salt degradation due to electrochemical reactions is very likely to be the cause. Cho et al. (2014) applied DC-powered electrochemical treatment on domestic wastewater, and they observed a 95% reduction of NH_4_^+^ after 6 h treatment. Part of the NH_4_^+^ was oxidized to N_2_, and lost its share of conductivity in the solution. The presence of chloride is essential to the ammonia removal in wastewater, and the chlorine/hypochlorite formed in electrolysis reactions can enhance the oxidation of NH_4_^+^ to a large extent (Chen et al., 2007, Li and Liu, 2009). Thus, the performance of electrical treatment with DC is partially dependent on sludge composition, as the electrical degradable ions will lose their share of contribution to the effective conductivity.

Many studies have reported salt concentrations in different types of wastewater, (Cornelissen et al., 2008, Knerr et al., 2011, Brandes, 1978, Halalsheh et al., 2008, Vlaeminck et al., 2009, Zamalloa et al., 2013), and the conductivity in some of these wastewaters may be sufficient for OH treatment. However, the waste sludge generally has much lower conductivity comparing to wastewater due to high solid contents. In addition, a large portion of the salts in human feces is made up of ammonium from urine, which may be subject to electro-chemical degradation as discussed above. Therefore, addition of salts may be required in order to initiate OH treatment. Different kinds of conductivity enhancers have been used in previous studies, including ammonium salts and organic sanitizers (Safaei et al., 2009, Takhtehfouladi et al., 2013, Habel, 2010). Many studies have demonstrated salt accumulation as a common issue in recycled water (Knerr et al., 2011, Wang et al., 2015, Leverenz et al., 2016, Benami et al., 2016). Since the onsite toilet system uses a close loop for water recycling for flushing, the salt concentration in the recycled water and sludge would be much greater than that commonly found in the wastewater treatment plants. These elevated salts although have potential negative impact for land application, it would facilitate OH treatment of sludge.

### 4.3 Pathogen inactivation in sludge

Comparing to conventional sludge treatment processes such as anaerobic digestion, electrical thermal treatment with optimized operational parameters is much more efficient in the aspect of pathogen inactivation. Previous studies showed that with assistance of organic sanitizer as conductivity enhancer, 11 log_10_ removal of reovirus and up to 8 log_10_ removal of *C. perfringens* were achieved in anaerobically digested biosolids within a few hours (Takhtehfouladi, 2007, Takhtehfouladi et al., 2013). Habel (2010) observed rapid and complete removal of *Ascaris suum* eggs by OH with the presence of ammonium nitrate. High removals of *Salmonella* spp. and fecal coliform in sludge as a result of electrokinetic treatment were also reported (Huang et al., 2008, Esmaeily et al., 2006). The voltage inputs of all these studies were < 5 V/cm.

In this study, > 6 log_10_ of *E. coli* were inactivated in < 2 h with the power input of 2 V/cm (18 V applied between two electrodes with gap of 9 cm) (Fig. 4). It should be noted that AC and DC treatment demonstrated different patterns of *E. coli* removal. When AC was applied, the reduction of *E. coli* occurred only when a “threshold temperature” (40 °C–50 °C) was reached. Comparatively, the temperature elevated slower with DC power, but the decay rate of *E. coli* was faster and steady during the course of the experiment. Cho et al. (1999) concluded that thermal effect was the primary mechanism for the inactivation of spores with OH. Other mechanisms in addition to thermal sterilization were involved during the sludge treatment with DC for the inactivation of *E. coli*. A number of short–lived and high–energy products formed in electro-chemical (EC) reactions have been suggested as the disinfection enhancers. Huang et al. (2016) demonstrated that chlorine generated during DC treatment was much more effective in disinfection of different kinds of pathogens than chemical chlorination. Hydrogen peroxide (H_2_O_2_) and chlorine dioxide (ClO_2_) are also common byproducts in electro-chemical reactions (Qiang et al., 2002, Bergmann and Koparal, 2005, Pillai et al., 2009), and they have been proven efficient in inactivating bacterial and viral pathogens (Huang et al., 1997, Lazarova et al., 1999, Labas et al., 2008, Juven and Pierson, 1996). In contrast, thermal disinfection due to OH was responsible for the inactivation of bacterial pathogen indicators during sludge electro-dewatering treatment reported by Navab-Daneshmand et al. (2012). This is because the sludge temperature in their study increased to 100 °C after a few minutes, and the microorganisms were sterilized shortly by such high temperature.

### 4.4 Applications of OH in sludge treatment

In the past decades, a number of innovative thermal methods were studied and applied for sludge treatment, including combustion, pyrolysis, gasification, and wet oxidation. However, in addition to the high capital investment of the infrastructure, these treatment processes also requires intensive energy inputs and professional operation and maintenance. They are generally not applicable in the decentralized and low resource environment. In contrast, OH, as a simple type of thermal treatment, has much lower energy demand and operational complexities, so it could be more favored in decentralized systems. The energy consumption of OH treatment in our study is comparable to the wastewater electrolysis cell described in Huang et al. (2016), to which solar energy is sufficient to serve as the sole energy source.

Attentions should be paid for the selection of power supply. DC power facilitates sludge dewatering (Fig. S2) and has greater potential for pathogen removal. However, the electrochemical reactions triggered by DC may impede the applications. In this study, we observed severe corrosion on the stainless steel electrodes after they were operated in sludge with 0.15 M NaCl at 2 V/cm of DC for 4 h. In contrast, corrosion with AC power was insignificant, that only a few dents were found on the edge of electrodes with same experimental condition. The corrosion may become a critical obstacle for the applications of DC power in sludge treatment, as being addressed in other related studies (Mahmoud et al., 2010, Mahmoud et al., 2011, Mahmoud et al., 2016, Citeau et al., 2012, Citeau et al., 2016). In addition, large amount of bubbles and foam were generated during DC treatment, and it may lead to operational issues in practice. If DC power is applied on electrodes made of stainless steel, replacement of electrodes may be required on weekly basis according to our experiments. Thus it may not be feasible and cost-effective especially in decentralized systems. Although a number of anti-corrosion electrodes, such as dimensionally stable anode (DSA) have been developed and commercialized (Kim et al., 2014), the costs of these electrodes are usually higher than the conventional ones, which may hamper the extensive implementations.

## 5 Conclusion

This study systematically investigated the EEC, energy efficiency, and pathogen removal using electrical thermal treatment for sludge disinfection. The ME-1 and EMT model were proven to be accurate for the prediction of the EEC. With proper operation parameters, the sludge temperature reached 70 °C within 2 h, in which no *E. coli* was detected after treatment. DC power offered higher *E. coli* removal even at lower temperature comparing to AC power. However, it led to severe corrosion on the electrodes, which may hinder long-term application. The temperature and conductivity were synergistic in ohmic heating process, and heat preservation is the key to promote energy efficiency.

## Funding

This work was supported by the Bill and Melinda Gates Foundation OPP 1111252.

## Appendix A Supplementary data

Supplementary material Image 1
