# Supplementary material for: Sludge disinfection using electrical thermal treatment: The role of ohmic heating
Source: Sci Total Environ. 2018 Feb 15;615:262–71. doi: 10.1016/j.scitotenv.2017.09.175 (PMC5711000; doi:10.1016/j.scitotenv.2017.09.175)
Supplement: Supplementary file 1 — Supplementary material [file mmc1.docx]

**Manuscript title:** Sludge Disinfection Using Electrical Thermal Treatment: The Role of Ohmic Heating

**Author:** Ziqiang Yin^†^, Michael Hoffmann^‡^, Sunny Jiang^†,^*

The number of pages: 3

Tables: 1 (Table S1)

Figures: 2 (Figures S1-S2)

**Supporting Information**

Summary of experimental and model parameters (**Table S1**)

Electric conductivity during OH treatment with different types of power supply and salt concentration (**Fig. S1**)

Loss of water percentage during OH treatment with different power supply and salt concentration (**Fig. S2**).

**Table S1.** Summary of experimental and model parameters

| Parameter | Symbol | Value |
| --- | --- | --- |
| Applied voltage | V | 18 V |
| Length of the reactor | A_s_ | 0.018 m^2^ |
| Initial pH of sludge mixture | -- | 5.5 – 6.5 |
| Initial weight of sludge mixture | m | ~ 0.1 kg |
| Thickness of the reactor wall |  | 0.005 m |
| Thermal conductivity of the wall |  | 0.2 W/m K |
| Specific latent heat of water evaporation | L | 2264.76 kJ/kg |
| Heat transfer coefficients in the reactor |  | 100 W/m^2^K |
| Heat transfer coefficients outside of the reactor |  | 10 W/m^2^K |


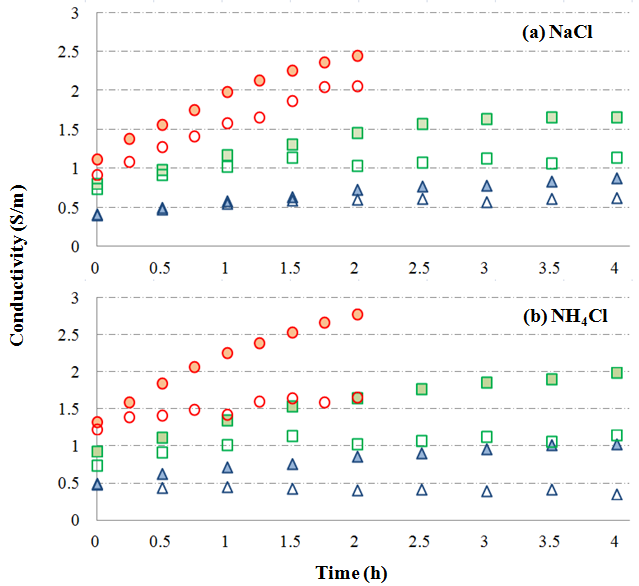


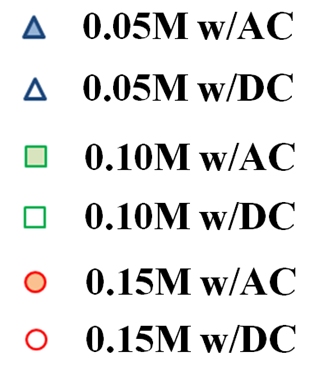


**Fig. S1.** Electric conductivity during OH treatment with different types of power supply and salt concentration


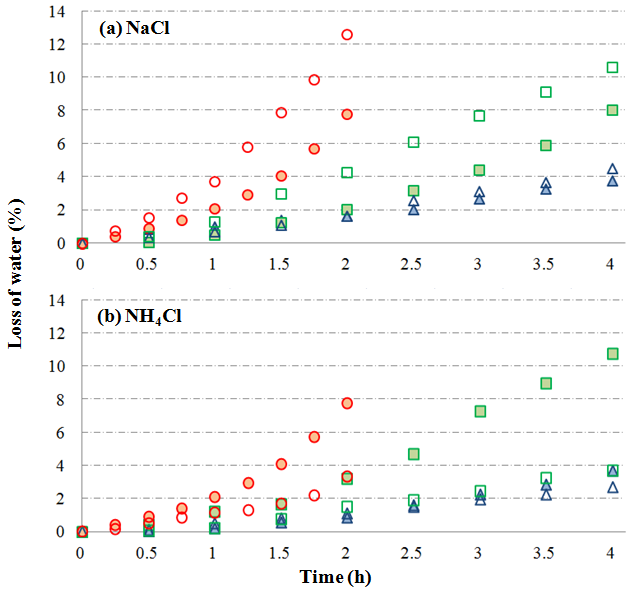


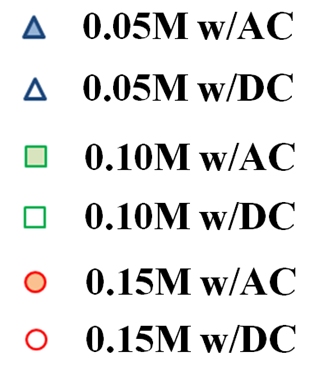


**Fig. S2.** Loss of water percentage during OH treatment with different power supply and salt concentration
